# HIV-1 Promoter Single Nucleotide Polymorphisms Are Associated with Clinical Disease Severity

**DOI:** 10.1371/journal.pone.0150835

**Published:** 2016-04-21

**Authors:** Michael R. Nonnemacher, Vanessa Pirrone, Rui Feng, Brian Moldover, Shendra Passic, Benjamas Aiamkitsumrit, Will Dampier, Adam Wojno, Evelyn Kilareski, Brandon Blakey, Tse-Sheun Jade Ku, Sonia Shah, Neil T. Sullivan, Jeffrey M. Jacobson, Brian Wigdahl

**Affiliations:** 1 Department of Microbiology and Immunology, Drexel University College of Medicine, Philadelphia, Pennsylvania, United States of America; 2 Center for Molecular Virology and Translational Neuroscience, Institute for Molecular Medicine and Infectious Disease, Drexel University College of Medicine, Philadelphia, Pennsylvania, United States of America; 3 Department of Biostatistics and Epidemiology, Center for Clinical Epidemiology and Biostatistics, University of Pennsylvania School of Medicine, Philadelphia, Pennsylvania, United States of America; 4 B-Tech Consulting, Ltd., Philadelphia, Pennsylvania, United States of America; 5 Center for Clinical and Translational Medicine, Institute for Molecular Medicine and Infectious Disease, Drexel University College of Medicine, Philadelphia, Pennsylvania, United States of America; 6 Division of Infectious Disease and HIV Medicine, Department of Medicine, Drexel University College of Medicine, Philadelphia, Pennsylvania, United States of America; University of Texas Rio Grande Valley, UNITED STATES

## Abstract

The large majority of human immunodeficiency virus type 1 (HIV-1) markers of disease progression/severity previously identified have been associated with alterations in host genetic and immune responses, with few studies focused on viral genetic markers correlate with changes in disease severity. This study presents a cross-sectional/longitudinal study of HIV-1 single nucleotide polymorphisms (SNPs) contained within the viral promoter or long terminal repeat (LTR) in patients within the Drexel Medicine CNS AIDS Research and Eradication Study (CARES) Cohort. HIV-1 LTR SNPs were found to associate with the classical clinical disease parameters CD4^+^ T-cell count and log viral load. They were found in both defined and undefined transcription factor binding sites of the LTR. A novel SNP identified at position 108 in a known COUP (chicken ovalbumin upstream promoter)/AP1 transcription factor binding site was significantly correlated with binding phenotypes that are potentially the underlying cause of the associated clinical outcome (increase in viral load and decrease in CD4^+^ T-cell count).

## Introduction

Numerous studies have identified human immunodeficiency virus type 1 (HIV-1) markers of disease progression/severity, with the majority being associated with viral load (VL), CD4^+^ T-cell count, host genetics, and immune responses. To date, VL and CD4^+^ T-cell counts have been the best markers of disease progression/severity and have long been used as prognostic markers of HIV-1 disease progression/severity [1–3]. Although these are good disease progression indicators, they are not thought to be predictive in nature. Recently, host genetic variants associated with clinical parameters have been discovered and validated by genome-wide association studies [4–9]. However, depending on the association being examined, there are also studies that do not show associations with certain clinical parameters like susceptibility or acquisition of HIV or T-cell response to certain vaccines [10–13].

Although many studies have examined the contribution of host factors to disease progression, few have focused on viral factors. The HIV-1 genotype and resultant phenotype are important variables of viral replication, which change during HIV-1 disease due to the low fidelity of the viral polymerase, inter- and intra-subtype recombination, rates of viral production, and host-specific selection pressures including G-to-A hypermutation caused by APOBEC (apolipoprotein B mRNA editing enzyme, catalytic polypeptide-like), antiretroviral drug resistance, immune system pressures, use of illicit drugs, and others [14–19]. HIV-1 genotypic variants are generated throughout HIV disease and are likely derived from a very small number of founder genotypes established in the earliest stages of infection from an initial swarm of viral quasispecies [20, 21]. During progressive HIV-1 infection, progeny viral swarms containing variant genomic sequences are continually produced [22] and many of these progeny viruses have broadened viral tropism and often increased cytopathic capability [16–18, 23, 24]. HIV-1 replication initially depends on the interaction of viral (gp120) and cellular entry proteins (CD4 and co-receptors, CXCR4 and CCR5) and subsequently on the regulation of viral gene expression driven by the HIV-1 long terminal repeat (LTR) from the integrated provirus. The LTR, in turn, relies heavily on participation of signaling pathways and cellular transcription factors (TFs) that can be modulated by external stimuli, as well as the viral transactivator protein Tat and viral regulatory protein viral protein R (Vpr), to guide viral gene expression [25–27]. Quasispecies development increases the complexity of regulated gene expression driven by the LTR, because TF binding sites (TFBSs) in the LTR may be altered functionally by the introduction of even single base-pair changes that may ultimately impact viral replication, potentially in a cell type- and tissue-specific manner [28–36], and may lead to compartment (including the brain)-specific differences in gene expression that may impact the HIV-1 disease course [37].

To date, few HIV-1 subtype B genetic variants have been associated with clinical parameters [30, 31, 35, 36, 38–40]. The HIV-1 envelope variant Asn283 (N283), which occurs in the CD4 binding site within gp120, has been found at a high frequency within brain samples derived from patients with HIV-1-associated dementia [38] and demonstrated the ability to decrease the gp120-CD4 dissociation rate, allowing for the use of lower levels of CD4 for viral entry as well as increasing viral replication in macrophages and microglia. We have been focused on genetic variation in the HIV-1 LTR and have previously shown in the pre-HAART (highly active antiretroviral therapy) era that patient-derived genetic variants in specific TFBSs correlated to HIV-1 disease severity and neurologic impairment [30, 31, 35, 36, 39]. Given these observations, the Drexel Medicine CNS AIDS Research and Eradication Study (CARES) Cohort in Philadelphia, PA, USA, was analyzed for clinical parameters associated with HIV-1 disease and viral single nucleotide polymorphisms (vSNPs) in the LTR were identified that are associated with decreased CD4^+^ T-cell count and increased VL and were consistent with functional alterations in the LTR that that have been shown to lead to increased viral cytopathic replication that would likely associate with increased HIV disease severity.

## Methods

### Ethics statement

The Drexel University College of Medicine Institutional Review Board (IRB) has approved this work under protocol 16311, which adheres to the ethical standards of the Helsinki Declaration (1964, amended most recently in 2008), which was developed by the World Medical Association as described [41]. All patient samples were collected under the auspices of protocol 16311 through written consent.

### Patient enrollment, clinical data, and sample collection

Patients in the Drexel Medicine CARES Cohort were recruited under protocol 16311 (Brian Wigdahl, PI), which adheres to the ethical standards of the Helsinki Declaration (1964, amended most recently in 2008), which was developed by the World Medical Association as described [41]. All patients provided written consent upon enrollment. Patients were called back for longitudinal study approximately every 6 months, with at least one recall per year as described in protocol 16311.

**Fig 1:**
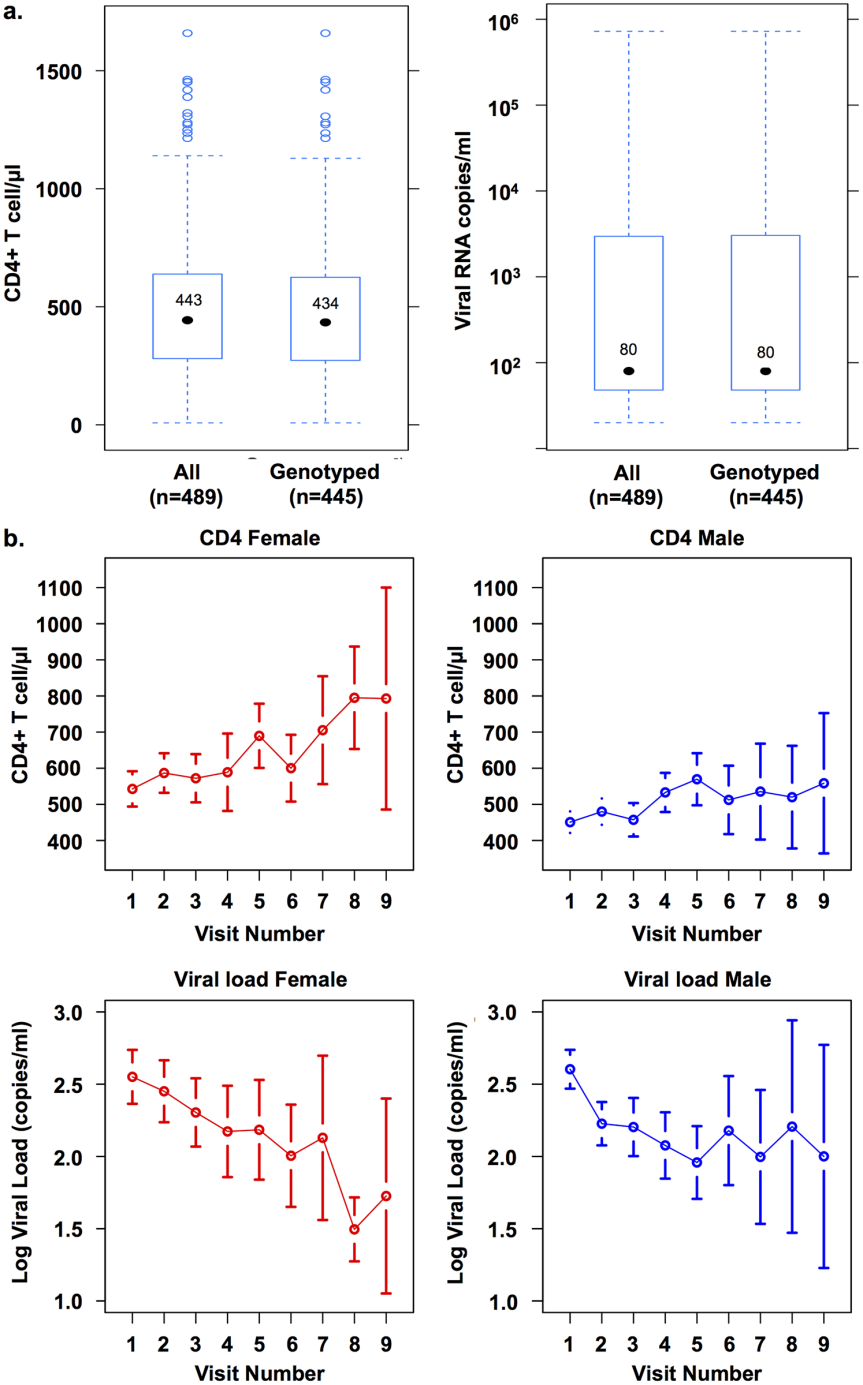
Distributions of CD4 and viral load (VL). (a) Boxplots of CD4^+^ T-cell count and VL of the 489 patients enrolled in the Drexel Medicine CARES Cohort at their screen visits and for the 445 patients who had their integrated HIV-1 proviral long terminal repeat (LTR) successfully amplified, sequenced, and viral single nucleotide polymorphisms (vSNPs) identified. The CD4^+^ T-cell count median was 443 with interquartile ranges (IQR) of 270 to 624 for the entire cohort and 434 with IQR of 266 to 624 for the genotyped individuals. The VL median was 80 with IQR of 48 to 3,233 for the entire cohort and 80 with IQR of 48 to 3,002 for the genotyped individuals. (b) Mean profiles of CD4^+^ T-cell count and log VL by gender and patient visit for the 445 genotyped patients.

The study reported here included 489 patients where 64.83% were male, 82.82% were black/African American patients, and 77.1% were on continuous HAART (Table 1). The average age was 45.1 years, with an average of 12.2 years of history since diagnosed as HIV-1 seropositive. The patients were followed longitudinally, with visits scheduled approximately every 6 months and were considered retained in the study as long as they were seen at least once a year. The average number of visits per patient was 2.755, and patients who had visited twice or more had a 66.8% retention rate. At the screen visit, the median CD4^+^ T-cell count was 443 interquartile ranges (IQR; 270–624) for the entire cohort and 434 (IQR; 266–624) for the genotyped individuals (Fig 1a). The VL median was 80 (IQR; 48–3,233) and 80 (IQR; 48–3,002) for the entire cohort and the genotyped individuals, respectively (Fig 1a). As expected, the distribution of VL was skewed and thus log transformation was used in later analyses. Among all patients, 91% had sequence available and these patients had characteristics (rightmost column in Table 1) similar to the entire cohort.

**Table 1:** Patient demographics at initial visit in the Drexel Medicine CARES Cohort of patients with HIV-1 LTR sequence.

Demographic Variables	Categories	Count (percentage)/Mean (±s.d.)	
		All (n = 489)	Genotyped (n = 445)
Gender	Male	317 (64.83%)	290 (65.17%)
	Female	166 (33.95%)	149 (33.48%)
Race	Black/AA	405 (82.82%)	372 (83.60%)
	White	57 (11.66%)	51 (11.46%)
	Other	17 (3.48%)	22 (4.94%)
Ethnicity	Hispanic/Latino	29 (5.93%)	26 (5.84%)
	Not Hispanic/Latino	459 (93.87%)	418 (93.93%)
	Unknown	1 (0.20%)	1 (0.22%)
Drug Use	Tobacco	353 (72.196%)	324 (72.81%)
	Alcohol	150 (30.67%)	133 (29.89%)
	Cocaine	370 (75.66%)	341 (76.63%)
	Cannabinoids	337 (68.92%)	311 (69.89%)
	Heroin	127 (25.97%)	116 (26.07%)
	Methamphetamines	71 (14.52%)	67 (15.06%)
	Benzodiazepines	64 (13.09%)	61 (13.71%)
	Narcotics	59 (12.06%)	56 (12.58%)
	Methylphenidate	20 (4.09%)	20 (4.49%)
HAART	Continuous	377 (77.10%)	345 (77.53%)
	Discontinuous	50 (10.22%)	46 (10.34%)
	Naïve	58 (11.86%)	50 (11.24%)
Age		45.1 (±8.6)	45.2 (±8.5)
Years Seropositive		12.3 (±7.1)	12.4 (±7.2)

“Other” includes Asian, American Indian/Alaskan Native, Native Hawaiian or other Pacific Islander, more than one race or unknown. HAART = highly active antiretroviral therapy.

### Peripheral blood mononuclear cell isolation

At each 6-month visit, blood was collected: One gray-top tube was sent for drugs-of-abuse screening (~10 mL) and four purple-top BD vacutainer tubes containing K2-EDTA as the anticoagulant (Becton Dickinson & Co., Franklin Lakes, NJ) were used to collect blood from patients (~40 mL) for serum and PBMC isolation, as described [41, 42]. From 5×10^6^ PBMCs, genomic DNA and total RNA isolation was performed using a Qiagen (Venlo, Limburg, Netherlands) AllPrep DNA/RNA procedure as described by the manufacturer.

### PCR amplification and sequencing of the HIV-1 LTR from patient genomic DNA

From the genomic DNA, PCR was performed to amplify and sequence the HIV-1 LTR as described [41]. Briefly, the first round of PCR was completed with two primers that are specific for the HIV-1 LTR (forward: 5'-TGGAAGGGCTAATTCACTC-3', reverse: 5'-ACTGATTTTCCCAGACTCCCT-3') (Integrated DNA Technologies [IDT]) along with Phusion High-Fidelity Polymerase (New England BioLabs, Ipswich, MA, USA), deoxyribonucleoside triphosphates (Promega, Madison, WI, USA), and magnesium chloride. From this first round of PCR, 10 μL of the reaction was used to complete a second amplification step using nested primers specific for the HIV-1 LTR (forward: 5’-CACTCCCAACGAAGACAAGA-3’, reverse: 5’-GAGGGATCTCTAGTTACCAG-3’) (IDT) and conditions similar to those in the first round. Following quantitation, the PCR product was purified using ExoSAP-IT (USB Corp., Cleveland, OH, USA) and was subsequently sequenced (Genewiz, South Plainfield, NJ, USA). Previous studies have demonstrated that analysis of RNA viral genomes for evidence of genetic variation within the genome may be affected by polymerase selection [43], where Taq polymerase was shown to create variants as a result of the polymerase rather than naturally occurring within the genome compared to *Pfu* DNA polymerase, which showed a greater fidelity and did not introduce false positive sequence variants. The studies presented here used the Phusion DNA polymerase that features an error rate 6-fold less than *Pfu* DNA polymerase, indicating that all variants detected in this study were the result of changes inserted by the patient’s viral polymerase and not a result of the amplification process.

PCR amplicons were then deep sequenced using an Illumina HiSeq as described by the manufacturer. Amplicons were purified and a library was made using the Nextera XT Library Prep procedure with the Nextera XT Index procedure v2 to produce the sequencing libraries and sequenced using the NextSeq 500 High Output v2, as described by the manufacturer. This typically produced 10 million paired end reads of approximately 150 nucleotides separated by an average insert size of 300 bp. A total of 384 samples were randomly selected from those previously described; of this set 269 samples had successful amplification and sequencing and were used for further study.

### Analysis of sequencing results

The overall LTR sequence for each patient was analyzed for sequence variation throughout the entire LTR as described [41]. Sequences were aligned to the Consensus B (Jan2002) reference sequence [44]. Both quality information from the trace files (PHRED scores [45]), and several statistical tests for identification and quality control of the called putative variations were used to identify high-quality vSNPs. Within the dbSNP database, the working definition of a SNP has been described as any change away from the reference genome and there are numerous poly-allelic examples. Given this, “vSNP” has been used here as a convenient abbreviated designation for any variant away from the ConB reference. The Neighborhood Quality Standard (NQS) method of Altshuler and Brockman was used for vSNP calling and validation [45, 46]. Final sequences have been submitted to Genbank under Bioproject ID PRJNA309974.

### Deep sequencing analysis

NGS sequences were first trimmed to remove adapters and PCR primers. They were then aligned to the ConB LTR using the BWA mem algorithm [47]. Only those reads in which both pairs mapped to HIV with a mapping quality greater than 60 were retained. The samtools mpileup algorithm [48] was used to count the frequency of each base at each position within the QS, taking into account the quality scores generated for each position. Final sequences have been submitted to the short read archive and all samples were linked under Bioproject ID PRJNA309974.

### Statistical analysis for identification of vSNPs associated with clinical disease parameters

Histograms and summary statistics of all variables were examined to understand selected aspects of data quality and to examine the normality assumption underlying statistical models. VL measurements exhibited a skewed distribution and were normalized through the base-10 logarithm transformation. The completeness and consistency of the genotype data were assessed and the mutation frequencies estimated. vSNPs sequenced from fewer than 23 samples (5% of the total) were removed due to unreliability. vSNPs that were monomorphic or had no mutation were considered uninformative and were filtered out.

### Model selection

In this study, analyses focused on CD4 T-cell count, VL, and longitudinal changes in these particular phenotypes. Considering gender, age, race, ethnicity, time from the first visit, selected drug use, alcohol consumption, and tobacco use as possible covariates, linear mixed models (LMM) were used [49] to investigate which demographic/environmental factors were associated with the interested phenotypes, while adjusting for within-individual correlations. A set of covariates was selected that were significantly associated with either CD4 T-cell count or log(VL) to be included in the vSNP—phenotype association models.

Gender, days since baseline visit, race, and age were significant predictors for either CD4^+^ T-cell count or VL (Table 2). Men had a lower CD4^+^ T-cell count than women *(P* = 0.0015); however, VL was not correlated with gender. As shown in Fig 1b, the mean CD4 counts of men and women steadily increased with each visit; however, the counts rose faster and to higher levels in women. A similar trend was observed with VL, which decreased over time, though decreases in women were greater and occurred faster. This may reflect the fact that most patients who remain associated with the study longitudinally have a greater tendency to take their antiretroviral medications. The reasons for these apparent gender differences remain to be determined. Days since screen visit and race were also significantly associated with an increase in CD4^+^ T-cell count and decrease in VL (Table 2). Interestingly, age was significantly associated with a decrease in VL but not in CD4^+^ T-cell count. Age at current visit was also highly correlated with days since the initial screen visit (Pearson correlation = 0.206, *P* = 4.32 × 10^−13^).

**Table 2:** Covariate analysis of patient demographic data associated with CD4^+^ T-cell count and log viral load measurements (Drexel Medicine CARES Cohort).

Phenotype	Variables	Level	Effect	*P* value
CD4	Gender	Female	Reference	-
		Male	-83.061	0.0009
	Age		-0.230	0.8675
	Days since screen visit		0.051	4x10^-7^
	Race	Black/African American	Reference	-
		White	-21.789	0.5582
		Other	103.847	0.0108
Log VL	Gender	Female	Reference	-
		Male	0.0144	0.8713
	Age		-0.015	0.0037
	Days since screen visit		-0.0004	3x10^-14^
	Race	Black/African American	Reference	-
		White	-0.270	0.0528
		Other	-0.313	0.0523

Only categories that had significant effects are shown. VL = viral load.

With these covariates now identified, each vSNP was coded according to whether it was a mutation, and if the mutation rate was greater than 5%, it was tested for association with phenotype (CD4 counts or VL) in LMMs, adjusting for the selected covariates. All significant vSNPs were tested further for association with the trend change in CD4 and VL over time by adding a vSNP—time interaction term in the LMM. To adjust for multiple comparisons in testing individual vSNP associations, the Benjamini-Hochberg method was used to control the overall false discovery rate at 0.05.

### vSNP sensitivity analysis of *in silico* binding prediction

A computational analysis was performed using the Jaspar position-weight-matrix (PWM) [50] for all TFs shown *in silico* to bind to the region spanning positions 98–132 in the HIV-1 LTR to investigate vSNP effects on TF binding to their cognate sites. The PWM from Jaspar measures the binding likelihood as a log-odds score in which lower values indicate a greater binding likelihood [51]. The potential effect on binding was examined using BioPython [52] to calculate the log-odds score for all possible vSNPs compared with ConB. The changes in log-odds score between the vSNPs and the ConB sequence at all positions were calculated. Larger values imply a binding increase. Using a χ^2^ test with 1 degree of freedom, a log-odds difference of 3.84 corresponds to a *P*<0.05.

### Electrophoretic mobility shift (EMS) analyses

Double-stranded DNA oligonucleotides corresponding to the ConB sequence were synthesized (Integrated DNA Technologies; Coralville, IA), with each of the four nucleotide derivations [LTR-A (ConB) = 5’ ACCAGGGCCAGGGATCAGAT 3’; LTR-T = 5’ ACCAGGGCCAGGG**T**TCAGAT 3’; LTR-C = 5’ ACCAGGGCCAGGG**C**TCAGAT 3’; LTR-G = 5’ ACCAGGGCCAGGG**G**TCAGAT 3’]. Oligonucleotides were gel purified and end labeled with radioactive [γ-^32^P] ATP (Perkin Elmer; Waltham, MA) by T4 polynucleotide kinase (Promega; Madison,WI). The binding reaction contained poly(deoxyinosinic-deoxycytidylic) (poly-dIdC; 1μg), 5X binding buffer (62.5% glycerol, 5 mM MgCl_2_, 750 mM KCl, 80 mM NaCL, 1 mM DTT, 50 mM Tris-HCL pH 8.0, 2.5 mM EDTA, 1% NP-40, and water), 10–20 μg of Jurkat and U-937 nuclear extract (Santa Cruz Biotechnology; Dallas, TX) was incubated on ice for 30 minutes before the addition of radiolabeled probe. Radiolabeled probe (75,000 cpm) was added to each binding reaction and incubated for an additional 30 minutes on ice. For supershift assays, 4 μg of antibodies (Santa Cruz Biotechnologies) directed against normal control rabbit IgG, COUP, ETS-1, GATA-2, c-Jun, and c-Fos were incubated with the binding reaction for 1 hour on ice before the addition of probe. Binding reactions were resolved on a 4.5% PAGE gel with 0.33X TBE buffer for 2.5 hours at 200 V. Gels were dried at 80°C by a Bio-Rad gel dryer and vacuum pump system for 2 hours. Phosphor screens were exposed to gel for 24 and 48 hours before analysis by autoradiography.

### Plasmid cloning and site-directed mutagenesis

LTR-conB108A and 108G sequences were synthesized and cloned into pGL3 by VectorBuilder (Cyagen Biosciences). LTR-LAI plasmids were cloned into the pGL3 luciferase expression vector (promega) as previously described [41, 53, 54]. LTR-LAI108 was mutagenized using GeneArt Site-Directed Mutagenesis System (Thermofisher) and the mutagenized product was transformed into DH5α cells and grown for 24 hours with appropriate antibodies. Colonies were picked and grown and plasmid DNAs were obtained utilizing a Miniprep procedure (Qiagen), quantified, and verified by Sanger sequence analysis (GeneWiz). The Miniprep plasmid preparations with the desired mutation were then used for large scale plasmid production using the Maxiprep procedure as previously described (Qiagen) to obtain sufficiently large quantities of plasmid DNA for transient transfection analyses.

### Transient transfection analyses

Both U-937 monocytic cells and Jurkat T cells were cultured as recommended by the American Type Culture Collection (ATCC) as described previously [41, 54]. Cells were subcultured (at a 1:2 dilution) at 24 hours prior to transfection. Cells were seeded in a 6-well plate with 2 mL of fresh media at a cell density of 1 X 10^6^ cells per ml and incubated at 37°C in 5% CO_2_ for 1 hour. Transfections were performed using X-tremeGENE HP DNA transfection reagent (Roche) as described by the manufacturer. Both cell types were co-transfected with 1 μg of experimental each experimental plasmid (LTR-conB and LAI) and 50 μg of a TK Renilla plasmid as an internal control. Cells were lysed 24 hours post transfection and a dual-luceriferase reporter assay was performed. LTR-108A (containing an A at position 108) for conB and LAI was set to a value of 1.0 and LTR-108G variants (containing an A-to-G change at position 108) were represented as fold change over LTR-108A. Three independent experiments were performed in triplicate and a representative experiment is shown. Error bars represent the standard deviation for a single representative experiment performed in triplicate.

## Results

### vSNP associations with clinical variables

PCR-amplified HIV-1 LTR products from 445 patients and 1,113 longitudinal samples were obtained to determine whether vSNPs present in the HIV-1 LTR correlated with clinical parameters of HIV-1 disease. Sequences were analyzed as described and SNPs were identified as compared with the ConB (2002) reference sequence, resulting in 14,180 putative vSNPs identified, and an additional 107 vSNPs that were heterozygous and could not be uniquely identified.

**Fig 2:**
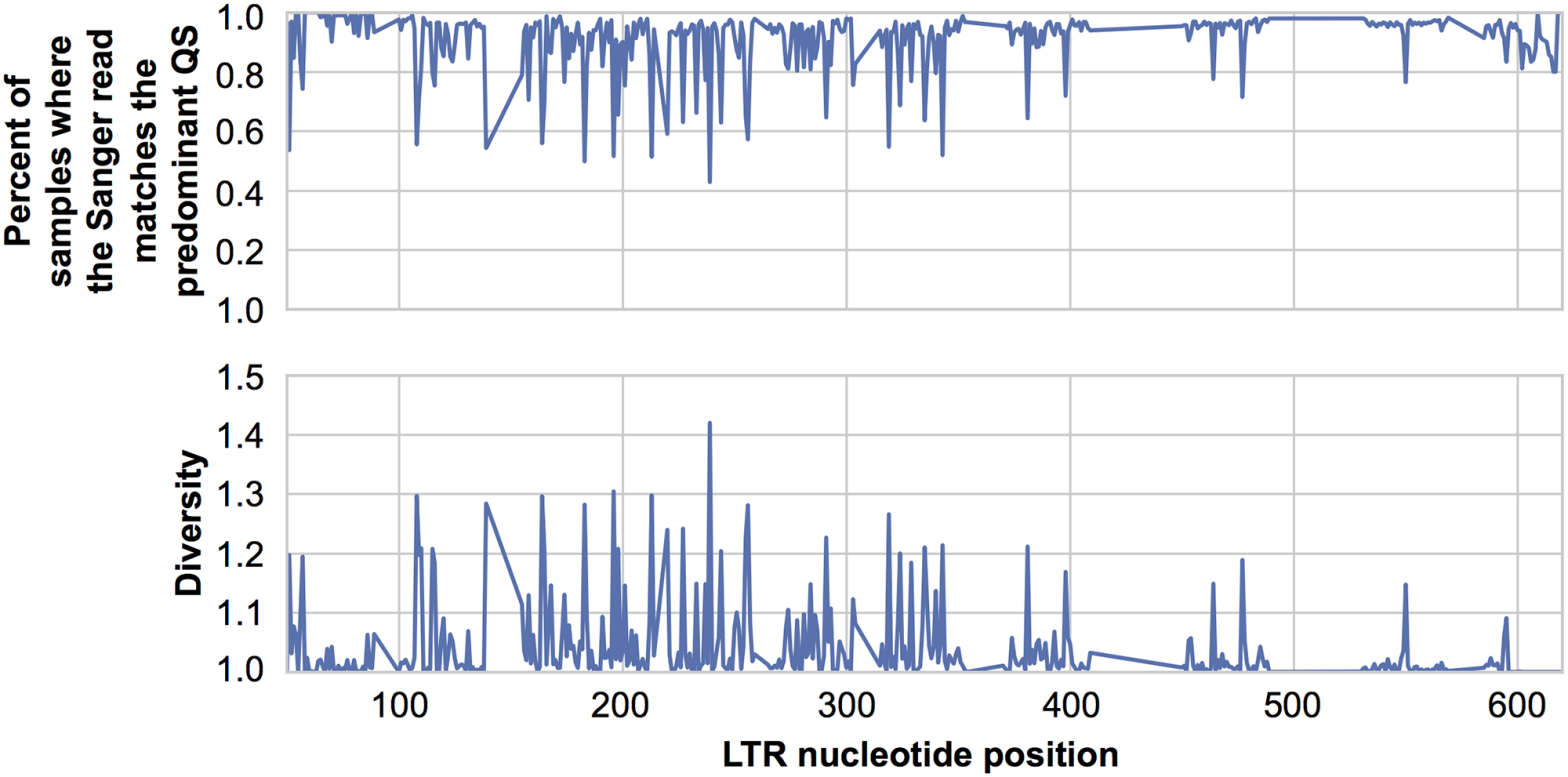
Deep sequencing confirms that Sanger sequenced PCR amplicons represent the predominant HIV-1 quasispecies. From the 1,113 longitudinal samples consisting of Sanger sequenced PCR amplicons 384 were randomly selected and deep sequencing of the LTR amplicon was performed. This resulted in 269 LTRs with quality sequence and at least 900X coverage. (a) The Sanger sequence was then compared to the deep sequences to determine the percent of samples where the two methods matched at each nucleotide. (b) The genetic diversity (order = 1) of each position of the LTR was calculated as previously described [55].

In order to determine whether the Sanger sequenced PCR amplicon represented the predominant sequence within a sample, a subset of sequences were examined using a deep sequencing approach. At any given nucleotide, the PCR read matched the predominant QS 91% [90.5–92.4 95% CI] (Fig 2a). Furthermore, by calculating the diversity [55], it was observed that most positions in the LTR consisted of a single variant (Fig 2b) with a mean diversity score of 1.037 [1.031–1.043 95% CI].

**Fig 3:**
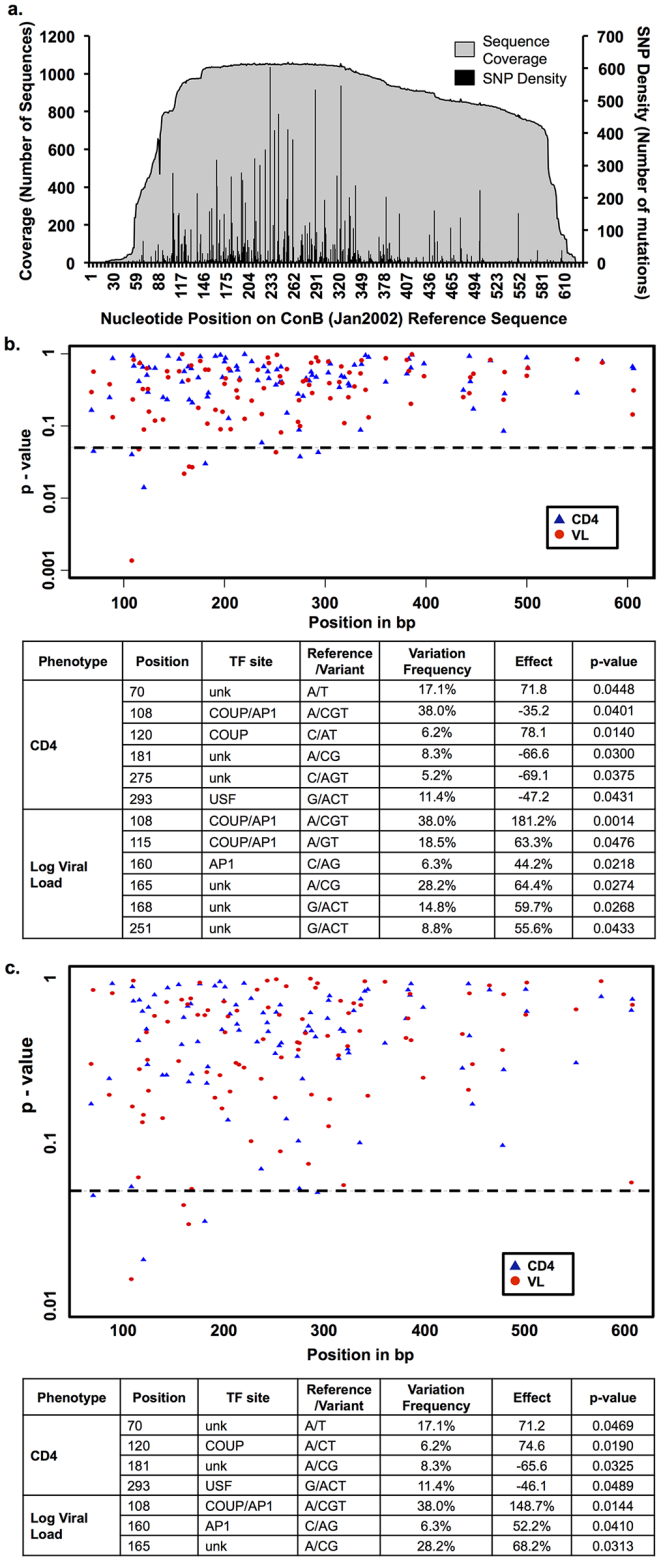
Viral single nucleotide polymorphism (vSNP) density and identification of vSNPs associated with CD4^+^ T-cell count and viral load (VL) within the HIV-1 LTR in the Drexel Medicine CARES Cohort. (a) Coverage and vSNP frequency across the HIV-1 LTR were determined for 1,113 LTR polymerase chain reaction sequences obtained to date by comparison to the ConB (Jan2002) reference sequence. Positional hotspots were identified at positions 108, 139, 164, 165, 183, 196, 198, 213, 220, 227, 233, 239, 244, 256, 262, 291, 319, 324, 335, 343, 381, 501, and 606. A position was considered a hotspot if >25% of the sequences contain a base other than the reference sequence. (b) Scatter plot and table of *P* values from individual SNP association tests for CD4^+^ T-cell count and log VL were determined using linear mixed models of the cross-sectional data adjusted for sex, age, race, and days since baseline visit. For each SNP, the position in the HIV-1 LTR was provided along with the ConB sequence nucleotide, the associated variation, the putative transcription factor binding site (TFBS), and the frequency at which this variation occurred within the cohort. The associated effect is provided where a minus sign is a decrease and the effect is measured away from the average. (c) Scatter plot and table of P values from individual SNP association tests for CD4^+^ T-cell count and log VL determined as in part “a,” but also adjusted for HAART (highly active antiretroviral therapy) status. Unk = unknown.

A vSNP density plot was generated and positional hot spots were identified (Fig 3a) and p-values for all single vSNP association results were obtained (Fig 3b). Six positional variations correlated with change in CD4^+^ T-cell count away from the average of the genotyped patients (468±281) and six positional variations correlated with change in VL away from the average of the genotyped patients (14,405±51,081) (Fig 3b). With regard to the CD4^+^ T-cell count, positional variations at 108, 181, 275, and 293 correlated with a significant decrease in CD4^+^ T-cell count while variation at positions 70 and 120 correlated with a significant increase in CD4^+^ T-cell count. All positional variations that associated with VL correlated with a significant increase in VL, with position 108 demonstrating the most dramatic difference with a 181.2% increase in VL when compared with the cohort average (Fig 3b). Interestingly, while a few of these vSNPs were contained in well-known TFBSs, many were not. For example, polymorphism in position 108 of the HIV-1 LTR lies in a previously characterized AP-1/COUP (chicken ovalbumin upstream promoter) TFBS [56, 57]. Analyzing the vSNPs with respect to a longitudinal change in the CD4 T-cell count over time reveals that position 108 remains significant with an effect of a loss of 0.181 CD4 cells/ml/day (66.1 cells/ml/year) with the mutation (q = 0.0416). Therefore, variation at position 108 correlates not only with an overall increase in viral load when compared to the cohort average, but also correlates with a decrease in CD4 T-cell count over time between patient visits.

This initial analysis was completed with corrections for possible confounders including gender, age, race, and time from baseline visit covariates. However, HAART status also has a strong potential to be a confounding factor, with patients within the cohort categorized as either continuous, discontinuous, or naïve to HAART (Table 1). Previous studies that examined mutation frequency prior to and after initiation of HAART from the gag-pol region of the HIV genome demonstrated that the rate of change during prior to initiation of HAART was estimated to be 50 to 70 nucleotide changes/10kb/yr, while genetic variation was reduced but still observed in patients on HAART to approximately 1 nucleotide change/10kb/yr [58]. However, this severely reduced rate may potentially be due to the fact that Joseffson et al. examined the gag-pol region of the genome from the memory T-cell compartment rather than across the entire peripheral blood mononuclear cell compartment. Given this observation and that HAART is known to decrease VL and increase CD4+ T-cell counts, the LMM was used with adjustments for HAART status in addition to the previous covariates (Fig 3c). With the addition of HAART as a potential confounding factor, changes at positions 70, 120, 181, and 293 remained correlated with a change in CD4^+^ T-cell count and changes at positions 108, 160, and 165 remained correlated with a change in VL, with all correlations maintaining the same directional effect, suggesting HAART therapy plays a role in selection of vSNPs in the LTR in conjunction with viral replication and perhaps other factors during HIV-1 disease. However, HAART did not eliminate the association observed with the majority of vSNPs and interestingly the strongest associations, positions 108 and 120, both lie within the COUP/AP1 region.

### Position 108 and the COUP/AP1 TFBSs

**Fig 4:**
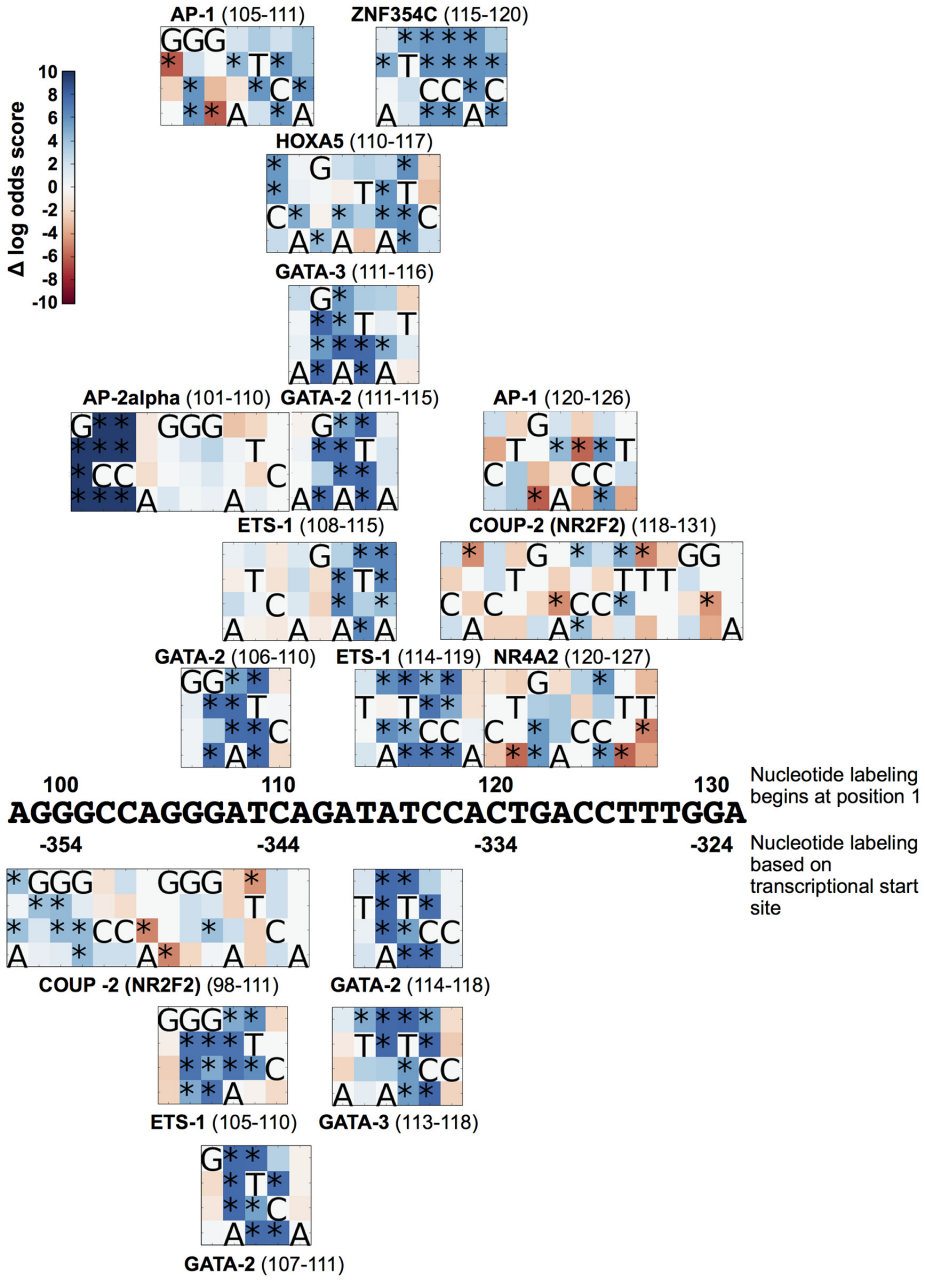
Viral single-nucleotide polymorphisms (vSNPs) at positions spanning 98–132 correlate with alterations in transcription factor binding potential. Heat maps representing the difference in log-odds score between the ConB sequence and all permutations of vSNPs for potential transcription factor binding between long terminal repeat (LTR) positions 98 and 132 are shown. The color indicates the negative delta log-odds score caused by the vSNP indicated in the y-axis and the position indicated by the x-axis, with higher values indicating an increased likelihood of binding. Changes were labeled as statistically significant, and marked with an asterisk, if the delta log-odds score was greater than 3.84, which corresponds to a *P*<0.05.

In the association analyses, variation at position 108 correlated with both a decrease in CD4^+^ T-cell count and an increase in VL in 38% of the patients (169), leading to the hypothesis that a variation at this site potentially increases viral replication at the expense of the CD4^+^ T-cell compartment, leading to an increase in HIV disease severity. Utilizing similar analyses, variation at position 108 associated with longitudinal changes in CD4 or VL, where the change was defined as the difference in CD4 or VL between the values at the visits and the baseline level at the screen visit (*P* = 0.0004 and 0.0216; data not shown), illustrating the potential importance of the COUP/AP-1 TFBS in this region. The importance of this region was further illustrated by the association of variation at positions 115 and 120 (Fig 3). Given this, an in-depth *in silico* analysis of this region was performed. Jaspar TFBS analysis of the region spanning positions 98–131 within the LTR revealed numerous TFs that can putatively bind within this region (Fig 4). Nucleotide sequence variation can enhance or diminish binding of particular TFs. Of particular interest was the enhanced binding profile demonstrated with GATA-2 and ETS-1, with any variation away from A at position 108, with a simultaneous decrease observed in COUP-2 binding. This does not diminish the potential that COUP-1 binding may increase; however, binding profiles and matrices are not available for COUP-1 within the Jaspar analysis. Similarly, variation at position 120 also demonstrated dysregulation of COUP-2 binding dependent on the variation observed, along with an increase in binding potential observed with NR4A2 and ZNF354C. Variation at position 115, which paralleled position 108 with a significant correlation between the variant and increased VL, correlated with increased binding potential of ETS-1, HOXA5, ZNF354C, GATA-2, and GATA-3.

### Electrophoretic mobility shift (EMS) assay and transient transfection analysis demonstrates the transcriptional effect of SNP108G

**Fig 5:**
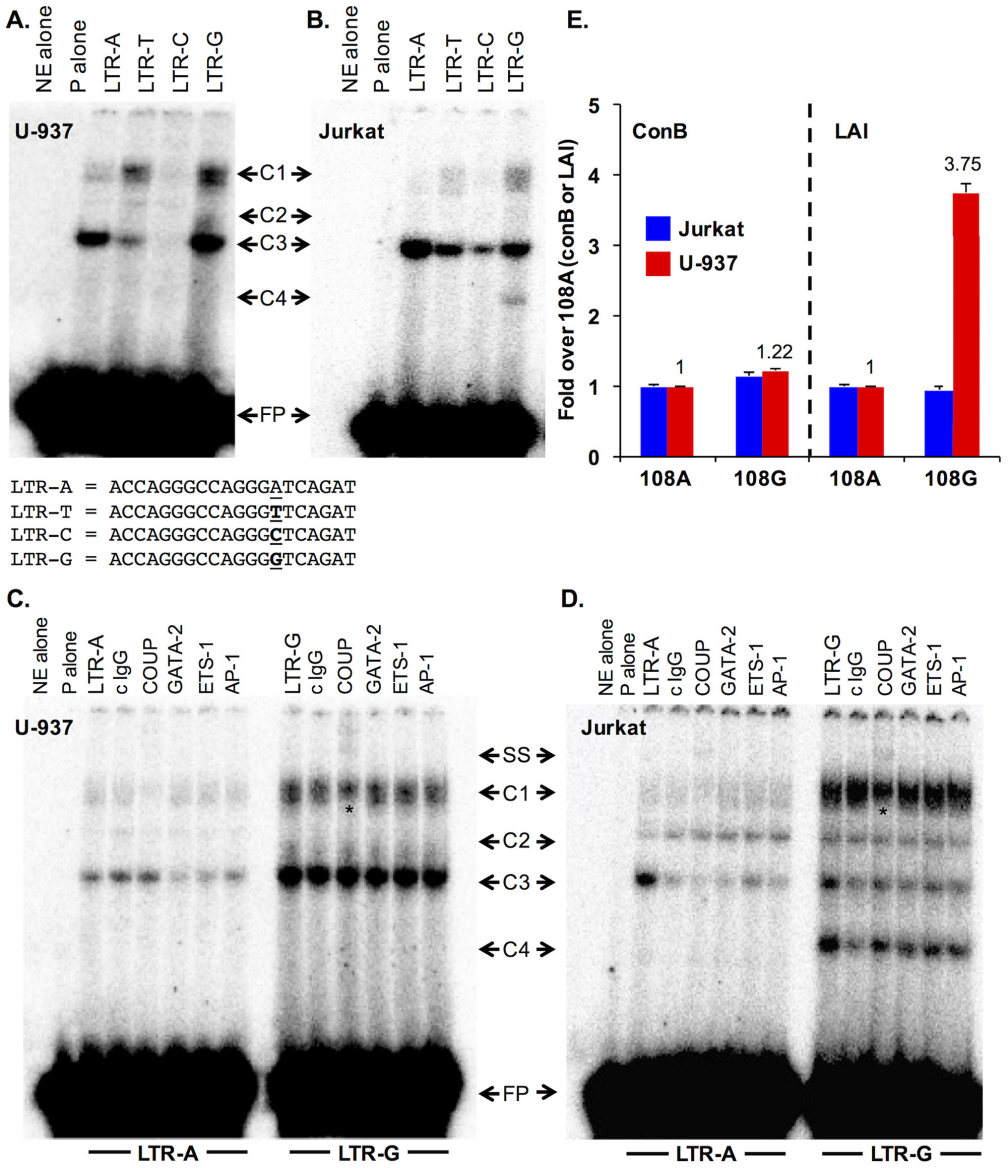
Electrophoretic mobility shift (EMS) assay and transient transfections demonstrates the transcriptional impact of vSNP 108. (a) EMS assay was conducted with ^32-^P radiolabeled oligonucleotides corresponding to each of the four base pair changes at position 108 and incubated with U-937 monocytic and Jurkat T-cell (b) nuclear extract. Reactions were conducted in excess of probe, indicated by free probe (FP) at the bottom of each gel. Complex formation (indicated as C1 to C4) occurred in the presence of nuclear extract and oligonucleotide but not in the presence of nuclear extract alone or probe alone. The sequence of the 4 oligonucleotides with each of the 4 base pair changes at position 108 is shown. (c) Oligonucleotide A (LTR-A) and G (LTR-G) were incubated with U-937 monocyctic and Jurkat T-cell (d) nuclear extract in the presence of control rabbit IgG antibody or antibodies against specific transcription factors (COUP, GATA-2, ETS-1, and c-Jun and c-Fos). COUP was supershifted (SS) in the presence of anti-COUP antibody and abrogated complex C1 as indicated by an asterisk (*). (e) The consensus B LTR and LAI LTR were cloned into the pGL3 luciferase expression vector to determine the effect of the A to G change at 108 on transcription in monocytic U-937 (red) and Jurkat (blue) T cells. Data represents a representative of three independent experiments conducted in triplicate and normalized to Renilla luciferase expression.

Four, 20 base pair long oligonucleotides spanning LTR position 94 (-360) to 114 (-340) with each of the indicated nucleotide changes at position 108 were synthesized to analyze the transcriptional significance of the A-to-G change in EMS analyses performed with U-937 monocytic and Jurkat T-cell nuclear extracts (Fig 5a and 5b). Direct transcription factor (TF)-DNA interactions were explored by EMS analysis to functionally validate *in silico* Jasper predictions with respect to relative affinities for each of the mutated oligonucleotide probes. Each of the four oligonucleotides were incubated with monocytic U-937 and Jurkat T-cell nuclear extracts and separated by a native PAGE gel. A change away from the consensus nucleotide A at position 108 (LTR-A) to any other nucleotide depicts a distinct TF binding profile (Fig 5a and 5b). When U-937 nuclear extract was incubated with LTR-A or LTR-G, three complexes were formed (C1 to C3), with varying intensities. When Jurkat nuclear extract was incubated with LTR-A, three complexes were formed (C1-C3). There was a significant increase in complex formation and TF binding, as well as the appearance of a new complex (C4) that occurred when position 108 was changed from an A-to-G; lending support to the Jasper analysis (Fig 4), which predicted overall increased TF binding in this region.

To discern the composition of the complexes formed, a supershift assay was conducted, whereby monocytic U-937 and Jurkat T-cell nuclear extracts were incubated with TF-specific antibodies prior to the addition of probe. Since 97% of the nucleotide changes at position 108 in the CARES Cohort were a change away from the consensus nucleotide A to G (data not shown), additional experiments were performed using only these two oligonucleotides. The binding profiles were determined for LTR-A and LTR-G with experimental antibodies directed against COUP (recognizes both the COUP I and II isoforms), GATA-2, ETS-1, c-Jun and c-Fos. When U-937 nuclear extract was incubated with LTR-A and LTR-G oligonucleotides alone, three complexes were formed with differing intensities as depicted (Fig 5c). With LTR-G alone, there was increased binding in C1 and C3 with abrogation of C1 and SS in the presence of COUP antibody. When Jurkat T-cell nuclear extract was incubated with LTR-A oligonucleotides alone, three distinct complexes were again apparent (Fig 5d). With LTR-G alone, a new, higher mobility complex (C4) was formed that wasn’t apparent when utilizing the probe for LTR-A. There was also a significant increase in TF binding as indicated by a lower mobility complex (C1), when compared to LTR-A. In both LTR-A and LTR-G, a supershifted COUP complex (SS) was apparent as well as a partial abrogation of low mobility complex (C1). Overall, supershift analysis confirmed the presence of COUP, GATA-2, EST-1, and AP-1 and the overall increase in TF binding when position 108 was changed from an A to a G in a cell-type dependent manner.

While the EMS analysis demonstrated differences in the TF binding profile between the monocytic U-937 and Jurkat T-cell types and begins to provide information relevant to the DNA-protein complex composition, additional studies are required to determine the effect of SNPs on the transcriptional activity of the LTR. As one experimental approach to examine the functional properties of nucleotide changes at LTR position 108, transient expression assays of conB and LAI LTRs bearing SNP 108 A or G driving luciferase expression further demonstrate the transcriptional impact of 108G SNP and supports the observed binding profiles determined by EMS assays (Fig 5e). LTRconB 108A was used as a baseline level of basal transcriptional activity since position 108 is an A in the LTR consensus sequence. When position 108 was changed to a G (108G), there was no significant impact on LTR activity in either U-937 monocytic or Jurkat T cells. Since the conB LTR and LAI LTR were identical in the 20 base pair region (-360 to-340) as the oligonucleotide used in EMS analyses, variants A and G at position 108 in LAI LTR backbone were analyzed to determine if the viral LTR would function differently than the consensus LTR. Similarly, the LAI 108A LTR was used as transcriptional baseline and a change from A-to-G at 108 showed a significant impact on transcriptional activity in U-937 monocyctic cells but not in Jurkat T cells, further supporting the cell-type dependent differences observed in EMS analyses.

## Discussion and Conclusions

The wide-spread usage of HAART has led to a decrease in overall mortality in patients with HIV-1 infection. Generally, patients treated with HAART remain healthier longer, with higher CD4^+^ T-cell counts and lower VL measurements. Conventionally, CD4^+^ T-cell counts and VL measurements were, and are still, used as a prognostic and diagnostic assessment tools with regard to the overall health of the infected patient; however, they are not predictive of other HIV-related and non-HIV-related events, such as neurocognitive impairment, and declines occur only in patients whose overall health is declining. Predictive markers indicating that a change in clinical disease course is imminent may therefore provide early opportunities for a change in the current therapeutic strategy or change in lifestyle. Clearly, immunologic, physiologic, or viral genetic markers may provide information to mitigate the potential decline in CD4^+^ T-cell count and increase in VL.

HIV-1 replication in lymphocytes and cells of the monocyte-macrophage lineage depends on regulation of viral gene expression driven by the LTR. The LTR, in turn, classically relies heavily on participation of cellular TFs, especially members of the nuclear factor kappa B (NF-κB), CCAAT enhancer binding protein (C/EBP) family, and Sp family, as well as the viral transactivator proteins Tat and Vpr, to guide viral gene expression [25–27]. Quasispecies development increases the complexity of regulated gene expression by the LTR, because TFBS in the LTR may be altered functionally by the introduction of even single base pair changes that may ultimately impact viral replication and potentially disease severity/progression. LTR sequence variation may play a role in tissue-specific disease or in the maintenance of viral reservoirs in particular cell populations during HAART. Numerous studies have reported sequence variation in LTRs isolated from patients with HIV-1 infection [28, 34, 59]. Changes within LTR sequences may also impact the ability of the LTR to support HIV-1 infection in different cell types by affecting binding sites for constitutive or cell type-specific TFs.

The Drexel Medicine CARES Cohort represents a unique longitudinal study of patients that can be assessed in the era of HAART for effects of variation within the viral genome itself in relation to clinical parameters indicative of overall health, including VL and CD4^+^ T-cell count. Analysis of the multiple variations within the LTR highlighted the importance of the nuclear receptor response element especially the COUP/AP1 TFBSs encompassing positions 108, 115, and 120 (Fig 3). Interestingly, previous studies performed on this TFBS showed that a change from an A-to-G at position 108 resulted in increased COUP binding [57, 60]. In the Drexel Medicine CARES Cohort, over 97% of the sequences with a variation at this position demonstrate an A-to-G change, which also results in an increase in TF binding. With respect to expression in cells relevant to HIV-1 infection, COUP has been found to be in T cells [56], monocyte-macrophages [61], astrocytes [56], and microglia [62]. Other studies have examined variations within this site and demonstrated that the A-to-G variation at position 108 increased binding of purified COUP; other studies confirmed this result [57]. Methylation assays have stressed the importance of positions 107 and 108 for protein binding [56, 57, 63], with studies indicating several mechanisms for both positive and negative regulation of gene expression [62, 64]. Within these studies, the change of position 108 from an A to a G showed in an in silico assessment that this change could have a potential effect on TF binding at several sites (Fig 4). In fact, these studies demonstrated that this change alters the intensity and number of DNA-protein complexes formed. In addition, it demonstrated that these complexes were different between T cells and cells of the monocyte-macrophage lineage (Fig 5).

It is also important to understand how these changes affect the general transcriptional rates of LTRs that contain the changes. This is important because, as stated above, COUP is a bifunctional TF that has been demonstrated to have both positive and negative effects on gene expression. On the same note, the cell type, neighboring TF binding partners, and coactivators/corepressors have been shown to greatly impact COUPs impact on LTR function. For example, the A-to-G change has been shown to enhance COUP binding, and it has been hypothesized to have a repressive effect on transcription and viral replication, due to its position within the negative regulatory element (NRE) [57]. Additional studies have shown that a 7 base pair mutation within the -350 to -327 region, which encompasses position -346 (108), increases transcription; however, others have demonstrated that a deletion of the region from positions -346 through -317 resulted in a decrease in transcription and replication rates, while deletions of other regions within the NRE resulted in enhanced transcription and replication in Jurkat T cells [60, 65]. Furthermore, within microglial cells, the presence of a G variant at position 108 resulted in increased binding of the COUP-TF along with increased LTR transcription [62] and these observations have been previously reviewed [66]. These observations suggest that A-to-G changes at position 108 within the COUP/AP1 binding site enhances transcription and acts as a transcriptional activator, suggesting a connection between the presence of the G variant and higher patient viral loads, which appeared to correlate with what has been observed within the CARES Cohort. To add to this, the results presented here demonstrate that HIV-1 transcription when position 108 has been altered from an A to a G depends on the LTR backbone as well as cell phenotype with the change in the LAI LTR demonstrating that 108G results in increased transactivation in Jurkat T cells. These results as well as previous studies by others help to explain differences in the transcription, replication rates, and TF-DNA complex formation when using selected sequences from a number of studies HIV-1 laboratory strains (LAI, JR-FL and HXB2) due to vSNPs in and surrounding position 108 [57, 62]. Many factors, including neighboring TFs, length between half-sites, affinity of TFs for specific binding sites, coactivators, and repressors all need to be considered in the overall impact of vSNPs on LTR function. Further analyses need to be performed to ascertain how the presence of position 108 as well as the presence of other vSNPs, both separately as well as in conjunction with position 108, impacts transcription and viral replication rates in several different LTR backbones. These studies are imperative with respect to understanding viral dynamics in this longitudinal cohort and from the viewpoints of viral latency and reservoir eradication efforts.

This study represents the largest study of LTR genetic variation to date and provides a unique approach involving the use of specific LTR vSNPs to associate with HIV-1 clinical parameters. Further studies will provide greater insights into the use of these viral molecular markers in prediction studies relative to disease severity. Future studies will also include expansion of these analyses across the entire viral genome to identify variants that are selected for or against during HIV disease that may lead to the identification of tools to predict the development of other HIV-1-induced clinical parameters. This work will in turn provide more information to guide the therapeutic management of HIV-1-infected patients. These studies will also provide a framework with respect to monitoring changes in the entire genome with respect to the use of Next Generation Sequencing strategies to assess relative changes in prevalence of specific nucleotides and amino acid residues with respect to changes in clinical severity as measured by changes in viral load and CD4^+^ T-cell counts or other clinical parameters.
